# Real‐time optical manipulation of cardiac conduction in intact hearts

**DOI:** 10.1113/JP276283

**Published:** 2018-08-07

**Authors:** M. Scardigli, C. Müllenbroich, E. Margoni, S. Cannazzaro, C. Crocini, C. Ferrantini, R. Coppini, P. Yan, L. M. Loew, M. Campione, L. Bocchi, D. Giulietti, E. Cerbai, C. Poggesi, G. Bub, F. S. Pavone, L. Sacconi

**Affiliations:** ^1^ European Laboratory for Non‐Linear Spectroscopy Florence 50019 Italy; ^2^ National Institute of Optics National Research Council Florence 50125 Italy; ^3^ Department of Physics University of Pisa Pisa 56127 Italy; ^4^ Division of Physiology, Department of Experimental and Clinical Medicine University of Florence Florence 50134 Italy; ^5^ Division of Pharmacology, Department ‘NeuroFarBa’ University of Florence Florence 50139 Italy; ^6^ R. D. Berlin Center for Cell Analysis and Modeling University of Connecticut School of Medicine Farmington CT 06030 USA; ^7^ Neuroscience Institute National Research Council Padova 35121 Italy; ^8^ Department of Biomedical Sciences Univercity ot Padua Padua 35121 Italy; ^9^ Department of Information Engineering University of Florence Via S. Marta 3 Florence 50139 Italy; ^10^ Department of Physiology McGill University Montreal Quebec Canada; ^11^ Department of Physics and Astronomy University of Florence Sesto Fiorentino 50019 Italy

**Keywords:** Optogenetics, Optical mapping, Digital Micromirror Device, Cardiac electrophysiology

## Abstract

**Key points:**

- Although optogenetics has clearly demonstrated the feasibility of cardiac manipulation, current optical stimulation strategies lack the capability to react acutely to ongoing cardiac wave dynamics.
- Here, we developed an all‐optical platform to monitor and control electrical activity in real‐time.
- The methodology was applied to restore normal electrical activity after atrioventricular block and to manipulate the intraventricular propagation of the electrical wavefront.
- The closed‐loop approach was also applied to simulate a re‐entrant circuit across the ventricle.
- The development of this innovative optical methodology provides the first proof‐of‐concept that a real‐time all‐optical stimulation can control cardiac rhythm in normal and abnormal conditions.

**Abstract:**

Optogenetics has provided new insights in cardiovascular research, leading to new methods for cardiac pacing, resynchronization therapy and cardioversion. Although these interventions have clearly demonstrated the feasibility of cardiac manipulation, current optical stimulation strategies do not take into account cardiac wave dynamics in real time. Here, we developed an all‐optical platform complemented by integrated, newly developed software to monitor and control electrical activity in intact mouse hearts. The system combined a wide‐field mesoscope with a digital projector for optogenetic activation. Cardiac functionality could be manipulated either in free‐run mode with submillisecond temporal resolution or in a closed‐loop fashion: a tailored hardware and software platform allowed real‐time intervention capable of reacting within 2 ms. The methodology was applied to restore normal electrical activity after atrioventricular block, by triggering the ventricle in response to optically mapped atrial activity with appropriate timing. Real‐time intraventricular manipulation of the propagating electrical wavefront was also demonstrated, opening the prospect for real‐time resynchronization therapy and cardiac defibrillation. Furthermore, the closed‐loop approach was applied to simulate a re‐entrant circuit across the ventricle demonstrating the capability of our system to manipulate heart conduction with high versatility even in arrhythmogenic conditions. The development of this innovative optical methodology provides the first proof‐of‐concept that a real‐time optically based stimulation can control cardiac rhythm in normal and abnormal conditions, promising a new approach for the investigation of the (patho)physiology of the heart.

## Key points

- Although optogenetics has clearly demonstrated the feasibility of cardiac manipulation, current optical stimulation strategies lack the capability to react acutely to ongoing cardiac wave dynamics.
- Here, we developed an all‐optical platform to monitor and control electrical activity in real‐time.
- The methodology was applied to restore normal electrical activity after atrioventricular block and to manipulate the intraventricular propagation of the electrical wavefront.
- The closed‐loop approach was also applied to simulate a re‐entrant circuit across the ventricle.
- The development of this innovative optical methodology provides the first proof‐of‐concept that a real‐time all‐optical stimulation can control cardiac rhythm in normal and abnormal conditions.

## Introduction

Closed‐loop stimulation is an experimental paradigm that enables new research and therapeutic targets by perturbing living tissue based on physiological parameters acquired in real time. In contrast to open‐loop experiments, which rely on the application of predetermined stimuli, closed‐loop experiments dynamically respond to living tissue, providing new insights into how these systems are controlled *in vivo*. Current applications in the cardiovascular sciences include dynamic clamp protocols which control electrophysiological properties in real time (Wilders, 2006; Altomare *et al*. 2015), the application of variably timed stimuli to control arrhythmias in intact tissue (Christini & Collins, 1996; Christini *et al*. 2006; Ploux *et al*. 2014), and protocols that create closed conduction circuits of variable length to explore the dynamics of cardiac re‐entry (Frame & Simson, 1988). Arrhythmia control strategies have first been demonstrated using microelectrodes for sensing and stimulating tissue, but these low‐resolution systems have a limited ability to respond to complex, spatially variable activity in cardiac tissue. More recently, researchers have investigated the use of optically captured signals to detect wave activity, which are then used to drive a stimulating electrode (Iravanian & Christini, 2007; Kulkarni & Tolkacheva, 2015). These approaches, however, cannot replicate the effect of a dynamically changing wavefront of conduction as the electrode is static. The emerging field of cardiac optogenetics offers a potential solution to this experimental problem.

Since its first application in cardiac sciences to map the origin of the cardiac pacemaker activity and precisely trigger the pacing rhythm in 2010 (Arrenberg *et al*. 2010; Bruegmann *et al*. 2010), optogenetic methods have been used for *in vitro* and *in vivo* research, ranging from single molecule studies to anti‐arrhythmic studies in the intact heart. A number of original studies and reviews have tried to validate optogenetics as a tool for clinical human applications (Ambrosi & Entcheva, 2014; Ambrosi *et al*. 2014; Vogt *et al*. 2015), while *in silico* simulations have established limitations and requirements for such a purpose (Boyle *et al*. 2013, 2015; Karathanos *et al*. 2016). The first therapeutic approach based on optogenetics was demonstrated in 2015 (Nussinovitch & Gepstein, 2015), where optogenetic resynchronization was performed employing a fibre‐optics‐based multi‐site illumination of a rat heart in which viral vectors were used for infection to express Channelrhodopsin‐2 (ChR2). Recently, three independent groups (Bruegmann *et al*. 2016; Nyns *et al*. 2016; Crocini *et al*. 2016a) have demonstrated one of the more expected therapeutic applications of ChR2: optical defibrillation. In this context, Crocini *et al*. 2016a explored new strategies of optical defibrillation using a patterned ChR2 stimulation based on previously acquired knowledge from optical mapping recordings. This approach limits the applicability of the optical manipulation within stationary dynamics. The possibility of optical manipulation of the electrical conduction based on ongoing cardiac dynamics may represent a new approach for the investigation of the physiology of the heart, opening promising perspectives for the clinical development of a minimally invasive optical pacemaker and novel resynchronization‐therapy devices.

Recently, a high‐resolution, all‐optical approach has been developed to achieve parallel optical control of cardiac excitation waves (Burton *et al*. 2015; Entcheva & Bub, 2016; Feola *et al*. 2017). Here, we closed the loop of truly parallel optical manipulation of cardiac activity by demonstrating an all‐optical, closed‐loop system comprising a high‐speed sCMOS camera as sensor combined with a digital micromirror device (DMD)‐based projector as the actuator. We controlled cardiac dynamics in genetically modified ChR2‐expressing mice by projecting user‐defined patterns at precise times on the myocardium based on detected cardiac activity. The system allowed real‐time intervention within 2 ms of detected activity, enabling tailored, tuneable modifications of cardiac dynamics.

## Methods

### Ethical approval

All animal handling and procedures were performed in accordance with the guidelines from Directive 2010/63/EU of the European Parliament on the protection of animals used for scientific purposes and conformed to the principles and regulations as described in the editorial by Grundy (2015). The experimental protocol was approved by the Italian Ministry of Health (protocol number 647/2015‐PR).

### Mouse model generation

As previously described (Zaglia *et al*. 2015; Crocini *et al*. 2016a), adult mice with a genetic background C57B6J and expressing cre‐recombinase under the control of the α‐MyHC promoter with B6.Cg‐Gt(ROSA)26Sortm27.1(CAG‐COP4*H134R/tdTomato)Hze/J (Charles River, Wilmington, MA, USA) were bred and the resulting offspring had the STOP cassette deleted in their heart, resulting in cardiac expression (α‐MyHC‐promotor driven) of the hChR2(H134R)‐tdTomato fusion protein.

### Isolated and perfused mouse heart

Transgenic mice were heparinized (5000 units mL^−1^) and anaesthetized by inhaled isoflurane (5%). The excised heart was immediately bathed in Krebs–Henseleit (KH) solution and cannulated through the aorta. The KH buffer contained (in mM): 120 NaCl, 5 KCl, 2 Mg_2_SO_4_, 1.8 CaCl_2_, 20 NaHCO_3_, 1.2 NH_2_PO_4_ and 10 glucose, then equilibrated with carbogen (95% O_2_ 5% CO_2_), pH 7.4. Contraction was inhibited with blebbistatin (5 μm) in the solution. The cannulated heart was retrogradely perfused (Langendorff perfusion) with the KH solution at 2–5 mL min^−1^ and then transferred to a custom‐built optical mapping chamber at a constant flow. Experiments were performed at 37°C with the exception of those relating to Figs 3 and 5 where, to reduce the conduction velocity, a temperature of 22°C was set. Two platinum electrodes were placed under the heart to monitor electrical activity via an electrocardiogram (ECG). After stabilization of the electrocardiogram frequency after a few minutes, 1 mL of perfusion solution containing the voltage‐sensitive dye (VSD; di‐4‐ANBDQPQ, 50 μg mL^−1^, University of Connecticut Health Center, Farmington, CT, USA) (Matiukas *et al*. 2007) was bolus injected into the aorta.

### Cell isolation and patch clamp recording

Ventricular cardiomyocytes from ChR2 transgenic mice were isolated by enzymatic dissociation as described before (Sacconi *et al*. 2012; Crocini *et al*. 2014b, 2016b; Scardigli *et al*. 2017). Briefly, animals are heparinized (5000 U kg^−1^, i.p.) and deeply anaesthetized with isoflurane. After excision, the heart was immediately bathed in cell isolation buffer and the proximal aorta was cannulated for perfusion in Langendorff mode. Buffer solution contained (in mM): 113 NaCl, 4.7 KCl, 0.6 KH_2_PO, 0.6 Na_2_HPO_4_, 1.2 MgSO_4_, 12 NaHCO_3_, 10 KHCO_3_, 10 Hepes, 30 taurine, 10 glucose and 10 2,3‐butanedionemonoxime, pH 7.3 (adjusted with NaOH). After perfusion at 37°C for 3–4 min with a constant flow of 7–8 mL min^−1^, the solution was switched to a recirculating enzyme solution, made from the same buffer with supplemented 0.1 mg mL^−1^ Liberase^TM^ (Roche Applied Sciences, Penzberg, Germany). After 7 min, the ventricles were excised and cut into small pieces in buffer solution supplemented with 1 mg mL^−1^ BSA. Gentle stirring facilitated further dissociation of myocytes. The cell suspension was left to settle and the cell pellet was resuspended in Tyrode buffer (in mm): 133 NaCl, 4.8 KCl, 1.2 MgCl_2_, 0.6 CaCl_2_, 10 glucose and 10 Hepes, pH 7.35 (adjusted with NaOH). Patch‐clamp studies were performed as previously described (Coppini *et al*. 2017). For resting membrane potential and action potential recordings, the pipette solution contained (in mM): 115 potassium methanesulfonate, 25 KCl, 10 Hepes, 3 MgCl_2_. Action potentials were elicited with short (3 ms) blue light pulses (470 nm) at a stimulation frequency of 2 Hz.

### Optical imaging and real‐time manipulation platform

Optical mapping and control were performed using a custom‐made mesoscope. The whole mouse heart was illuminated in wide‐field configuration using a ×2 objective (TL2x‐SAP, Thorlabs, Newton, NJ, USA) and a light emitting diode (LED) operating at a wavelength centred at 625 nm (M625L3, Thorlabs) followed by a band‐pass filter at 640/40 nm (FF01‐640/40‐25, Semrock, Rochester, NY, USA). The heart was illuminated with a maximum intensity of 1 mW mm^−2^. A dichroic beam splitter (FF685‐Di02‐25 × 36, Semrock) followed by a band‐pass filter at 775/140 nm (FF01‐775/140‐25, Semrock) was used for collecting the VSD‐emitted fluorescence. Notably, the tdTomato fusion protein co‐expressed with ChR2 was not excited at this excitation wavelength. A ×20 objective (LD Plan‐Neofluar ×20/0.4 M27, Carl Zeiss Microscopy, Oberkochen, Germany) was used to focus the fluorescence in a central portion (128 × 128 pixels) of the sensor of a sCMOS camera (OrcaFLASH 4.0, Hamamatsu Photonics, Shizuoka, Japan) operating at a frame rate of 1.6 kHz (630 μs actual exposure time). To manipulate electrical activity, a Texas Instruments Lightcrafter 4500 projector (Dallas, TX, USA), which contains a digital micro‐mirror device (DMD), and three high‐powered LEDs, enabled projection of user‐defined light patterns onto the heart. The projector is optically integrated with the mesoscope using a high numerical aperture (NA; 0.1) relay system providing a high coupling efficiency of the order of 30%. Importantly, the Lightcrafter's LEDs were driven by an external driver (LM3433‐1 LED Driver Evaluation Board, Texas Instruments) to achieve higher light intensities. Both green and blue emission were exploited in our experiments, using a second dichroic beam splitter (FF484‐FDi0136, Semrock) for the ChR2 spectral activation range. All microscope components were fixed onto a custom vertical honeycomb steel breadboard. See the Appendix describing exhaustively all necessary optical, mechanical and electronical specification and details to reproduce the system.

### Data analysis

Δ*F*/*F*_0_ imaging of electrical activity was performed using ImageJ: for each frame, the mean baseline was first subtracted and then the frame was normalized to the mean baseline yielding a percentage change in fluorescence over time. Since the blue light employed for ChR2 stimulation could affect recorded traces by exciting the VSD, where appropriate, the single frame containing a locally confined stimulation artifact was removed during post‐processing. The activation maps were generated by performing a threshold operation (pixel intensity >50% maximum) on the cross‐correlation data sets and replacing the active pixels with a time stamp. Sequential frames were collapsed into one frame where each pixel corresponds to the time delay of activation with respect to a user‐defined start time. Isochronal lines are found using a marching squares algorithm, and colour coded using a blue lookup table. For patch clamp experiments, the electrical data were analysed using pCLAMP (Molecular Devices, Sunnyvale, CA, USA).

### Statistics

**Figure 1:**
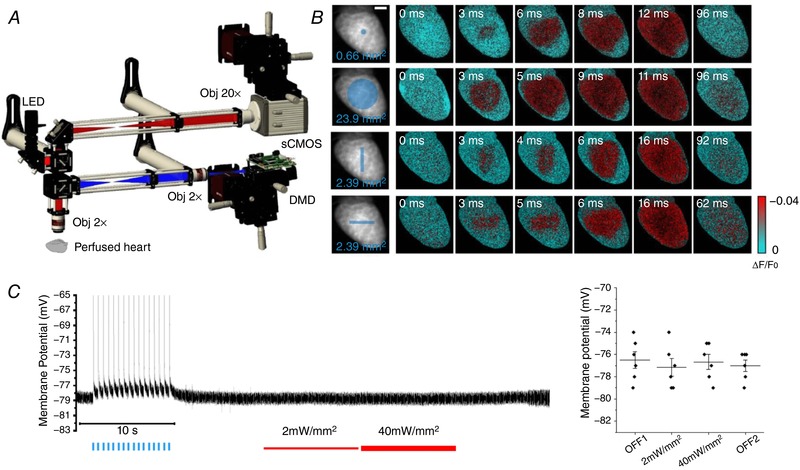
Targeted optogenetic manipulation of cardiac conduction *A*, scheme of the wide‐field fluorescence mesoscope. A red LED followed by a band‐pass filter (640/40 nm) excites through a ×2 objective the whole mouse heart, stained with a red‐shifted electro‐chromic voltage‐sensitive dye (Di‐4‐ANBDQPQ). A dichroic beam splitter followed by a band‐pass filter (775/140 nm) is used for collecting the emitted fluorescence signal. A 4f system is adopted to collimate the beam onto a ×20 objective. The signal is then focused into a central portion (128 × 128 pixels) of a sCMOS sensor operating at a frame rate of 1.6 kHz (630 μs actual exposure time). A commercial light steering solution based on a digital micro‐mirror device (DMD) was coupled with the mesoscope using a high numerical aperture relay system and a dichroic beam splitter. *B*, optical mapping during four patterns of optogenetic stimulation: single‐point, whole ventricle, vertical line and horizontal line, designed as reported by the blue traits on the fluorescence image (*F*_0_) of the heart. Six representative frames of optical mapping (∆*F*/*F*_0_) showing the electrical activation in red and the baseline in cyan. All stimulations were performed with a light intensity on the sample of 4 mW mm^−2^ and 2 ms exposure time. Scale bar: 2 mm. *C*, on the left, a representative trace of membrane potential recorded in cardiomyocytes isolated from ChR2 heart using patch clamp. At the beginning of the recording (OFF1), the optogenetic excitability of the cell was confirmed inducing sixteen action potentials using blue light pulses (blue lines). In the remainder of the recording, the red LED used for VSD imaging was turned on with two different power intensities (2 mW mm^−2^, thin red line and 40 mW mm^−2^, thick red line) and turned off again (OFF2) showing no variation in resting membrane potential. On the right, graph shows resting membrane potential during different states of red LED illumination. Each data point (black circle) represents the average of membrane resting potential of a different cell. Also indicated is the mean and the standard error of the mean of each illumination state. No statistically significant differences (ANOVA; number of mice, 2; number of cells, 6) were found between categories.

To demonstrate the robustness of the presented method, three hearts were used for each experimental category (Figs 3, 4, 5). The robustness was validated by the stability of the closed‐loop in tens of trials and the reproducibility of the imposed real‐time manipulation (5–10 trials per heart per experiment). Data are expressed as means ± SEM. Patch clamp experiments (Fig. 1 *C* and Supporting information Fig. S1) were performed using six cells of two mice. Analysis of variance (ANOVA) was used between the four experimental classes (OFF1, 2 mW mm^−2^, 40 mW mm^−2^, OFF2). Dose–response curve experiments (Supporting information Fig. S2) were performed using six mice. In this experiment, the activation trials were analysed using binomial distribution statistics where the Wilson score interval was applied.

## Results

A wide‐field mesoscope (Fig. 1 *A*) operating at 1.6 kHz was used to map the action potential propagation in Langendorff horizontally perfused mouse hearts stained with a red‐shifted VSD (di‐4‐ANBDQPQ; Matiukas *et al*. 2007). The platform, implemented with a DMD‐based light crafter, enabled projection of user‐defined light patterns onto the heart. We used transgenic mice expressing the photosensitive ion channel ChR2 in the myocardium under the control of the α‐MyHC promoter and exploited light‐induced depolarization to manipulate local electrical functionality (Fig. 1 *B*). Notably, the use of a VSD excitation wavelength of 640 nm completely avoided ChR2 excitation (excitation peak at 470 nm) allowing patterned optogenetics stimulation during optical mapping. Specifically, using patch‐clamp experiments on isolated ChR2‐expressing cardiomyocytes, we found that during red light illumination (even applying 40 times stronger intensity than necessary for imaging) there was no significant variation of resting membrane potential (Fig. 1 *C*) and action potential amplitude or duration (Supporting information, Fig. S1).

The response of the heart to different ChR2 patterns in terms of illumination intensity and pulse duration (dose–response curves) was characterized (Supporting information Fig. S2). We found that the platform allowed elicitation of action potential even with a very small excitation area, opening the possibility of pattern stimulation at high spatial resolution.

**Figure 2:**
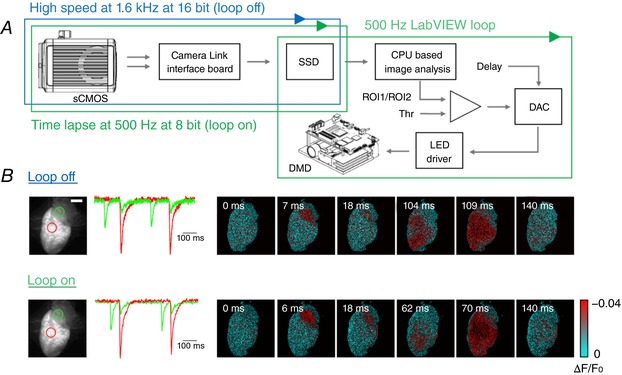
Real‐time optical intervention *A*, schematic workflow of the hardware and software architecture. A sCMOS camera, connected through two camera links to the workstation, saves the data in a RAID array of solid‐state disks (SSD). The camera can run either in high‐speed (blue cycle) or time‐lapse modality for closed‐loop operation (green cycle). During the latter, custom‐written LabVIEW software performs a real‐time analysis on images as they are saved on the SSD (green cycle): a previously acquired image of the heart is used as reference to select two ROIs, whose mean values are compared every 2 ms. When the ratio between the two ROIs exceeds the absolute value of a set threshold (Thr), optogenetic stimulation is activated, with user‐defined temporal delay and intensity. *B*, optical mapping during sinus rhythm with the two modalities: in loop‐off at a frame rate of 1.6 kHz (16‐bit images) and in loop‐on modality operating at 0.5 kHz (8‐bit images). The fluorescence signal (∆*F*/*F*_0_) of two ROIs selected on atrium and ventricle (green and red circle respectively, reported on the fluorescence baseline image) are shown for both acquisition modalities. Scale bar: 2 mm.

The system allowed two operating modalities: a loop‐off mode (i.e. free‐run optical mapping with the capability to illuminate at predefined intervals without active control) with a temporal resolution of 630 μs, producing 16‐bit images (Fig. 2 *A*, blue cycle), and a loop‐on mode (closed‐loop), with a temporal resolution of 2 ms, producing 8‐bit images. In loop‐on mode (Fig. 2 *A*, green cycles), while HC Imaging software (Hamamatsu Photonics) acquired and saved images in time‐lapse modality in a solid‐state disk (SSD) at 500 Hz, custom‐made LabVIEW software performed real‐time image analysis of the saved images. The fluorescence signal from either a single region of interest (ROI) or else the ratio from two distinct ROIs was calculated at every cycle and the last obtained value was compared with the already processed ones: when the current value overcame a set threshold, the software activated the DMD stimulation with an imposed temporal delay and illumination exposure. Even though computationally more intensive, the ratiometric approach can be used in photobleaching‐sensitive experiments. The performance of the imaging set‐up in both loop‐off and loop‐on modes was evaluated during sinus rhythm in Langendorff perfused hearts: Fig. 2 *B* shows cardiac electrical action potential propagation, which could be resolved temporally and spatially even in the lower‐resolution loop‐on modality.

**Figure 3:**
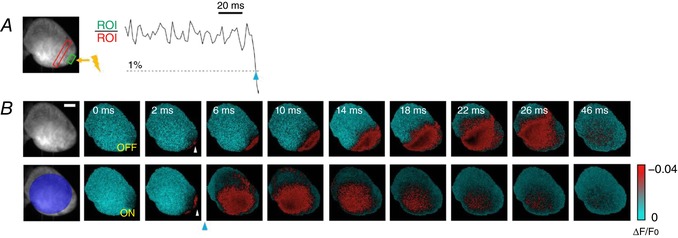
Intra‐ventricular manipulation *A*, a slowing down of the ventricular action potential propagation was induced by applying an electrical stimulation on the apex of the ventricle (yellow lightning). Two reference ROIs were selected (red and green rectangles) and the ROI ratio was obtained, with a 1% threshold and with no delay in activation. ChR2 stimulation was achieved with a light intensity of 4 mW mm^−2^ and 2 ms exposure time (blue arrowhead). *B*, the wave‐front propagation without the real‐time intervention (OFF) and with the optogenetic activation (ON). Frames acquired after photo‐stimulation (blue arrowhead) clearly show the differences in wave‐front propagation, highlighting the acceleration of ventricle activation. Scale bar: 2 mm.

First, we validated real‐time cardiac intervention in paced (control) conditions. Electrical (ECG) and optical recordings were used to monitor spontaneous activity. Using a bipolar electrode, a burst of electrical stimuli (3 ms in duration at 5 Hz) was delivered to the heart apex (yellow arrow in Fig. 3 *A*) to overdrive the sinus rhythm. Without optical stimulation, VSD mapping of electrically stimulated activity demonstrated uniform propagation from the apex to the surrounding myocardium (Fig. 3 *B*, top row). Using the ratiometric approach, two ROIs were selected near the apex as shown in Fig. 3 *A* (green and red rectangles). When their ratio crossed the threshold, the projector illuminated the ventricles with a large circular pattern (Fig. 3 *B*, bottom row). Considering a typical shot noise at rest within our ROI (typically of an area of 200–600 pixels) of 0.2–0.02% of  Δ*F*/*F*_0_ and VSD sensitivity of ∼4% (see Supporting information Fig. S3), a threshold of 1% of  Δ*F*/*F*_0_ was selected. The electrical stimulus generated a wave that initially (0–2 ms) propagated following the same kinetics shown in loop‐off mode but, as shown in the frame acquired at 6 ms, the optogenetic manipulation immediately activated the whole ventricle anticipating the electrical propagation. On average, experiments performed in three different hearts (total number of trials = 21) displayed a whole ventricle activation time of 27.8 ± 0.7 ms in loop‐off mode *versus* 4.7 ± 0.3 ms during closed‐loop optical manipulations.

**Figure 4:**
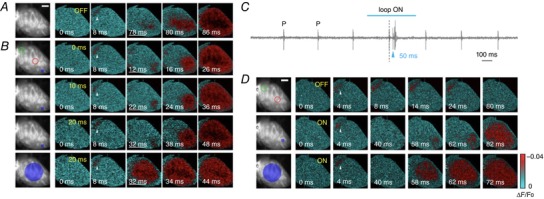
Optical manipulation of atrioventricular delay *A*, optical mapping of spontaneous heart activity, with typical atrioventricular (AV) temporal delay around 70 ms. *B*, the optical system was used to reduce AV delay by triggering the optical stimulation of the ventricle according to optically mapped atrial activity. The two reference ROIs (red and green circles) are positioned on atrium and ventricle respectively (1% threshold). As first stimulation pattern, a single point (blue area) was applied on the ventricle with three different temporal delays: 0, 10 and 20 ms after atrium activation. The underlined time frames highlight different delays. In the last row, whole‐ventricle activation was performed with a temporal delay of 0 ms. ChR2 stimulation was achieved with a light intensity of 4 mW mm^−2^ and 2 ms exposure time. White arrowheads indicate atrial activation. Scale bar: 2 mm. *C*, a stationary AV block was established by adding ethanol to the perfusion solution. ECG clearly demonstrates the presence of an AV block, since the P wave is not followed by a proper QRS complex. *D*, after inducing an AV block, the OFF modality shows no ventricular activation after atrial triggering. A real‐time intervention (ON) was performed using single‐point and wide‐area illumination, with 50 ms delay (1% threshold, light intensity of 4 mW mm^−2^ and 2 ms exposure time). Photo‐activation results in AV resynchronization, restoring normal cardiac conduction patterns during sinus rhythm. White arrowheads indicate atrial activation. Scale bar: 2 mm.

We then evaluated the system's ability to control ventricular activation time after detection of atrial activity. The system was first applied to manipulate the atrioventricular (AV) node delay that under sinus rhythm was found to be of the order of 70 ms (Fig. 4 *A*). In this experiment, the two ROIs were positioned on the atria (Fig. 4 *B*, green circle) and ventricle (Fig. 4 *B*, red circle), and the stimulation threshold was set to 1% of  Δ*F*/*F*_0_. The anticipation of the ventricle activation was performed imposing a user‐defined delay of 0, 10 and 20 ms between the trigger and the DMD stimulation (Fig. 4 *B* and Supporting information Fig. S4). The ventricle activation was induced projecting a localized spot at the apex or a wide circle of light (Fig. 4 *B*, blue areas). A similar approach was also used to restore timely ventricular activation after an AV block. Here, we experimentally induced AV block by adding 50 μL of ethanol to the perfusing solution, which resulted in a progressive loss of coordinated activity between the atria and ventricles. We monitored live ECG recordings to confirm complete AV block before starting optical stimulation (Fig. 4 *C*). VSD mapping clearly showed no ventricular activation after atrial activity (Fig. 4 *D*, top row). The system then projected a localized spot (Fig. 4 *D*, middle row) or wide circle of light (Fig. 4 *D*, bottom row) on the ventricle after user‐defined delays of 50 ms after detection of atrial activity. Repeated experiments performed in three different hearts (total number of trials = 16) showed a 100% successful capture of optogenetic ventricular activation following AV block for different stimulation patterns. These proofs of principle clearly demonstrate the ability of our methodology to manipulate the onset and the propagation properties of the action potential across the ventricle and AV resynchronization after block.

**Figure 5:**
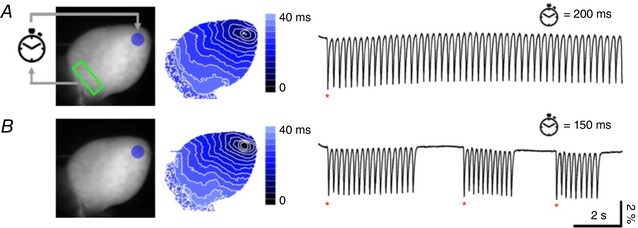
Optically induced re‐entrant circuit Left, fluorescence image of the heart. Re‐entrant circuit was started by detection of spontaneous ventricular activity at the base of the heart (green ROI). After detection, ChR2 stimulation at ventricle apex (blue circle) was applied after a delay of 200 ms (*A*) or 150 ms (*B*) concluding one loop‐of the cycle. Scale bar: 2 mm. Centre, corresponding colour‐scaled isochronal map reporting the activation time per pixel. Right, fluorescence signal (∆*F*/*F*_0_) extracted from the green ROI. The stable re‐entrant circuit at a stimulus delay of 200 ms (*A*) becomes unstable at a delay of 150 ms (*B*) showing discontinuity in the action potential time interval. Spontaneous action potentials (red asterisks) detected in the green ROI are needed in this case to re‐start the re‐entrant cycle.

As a final example, the system was used to mimic a pathological condition by simulating a re‐entrant circuit in a healthy heart. The system initiated with the detection of spontaneous activity (marked with a red asterisk in Fig. 5) within a continuously monitored ROI placed in the base of the ventricle. Once detected, the system applied an optogenetic trigger to generate an action potential at the apex of the heart. The action potential then propagated toward the base and once a variation in VSD fluorescence intensity was again detected within the ROI, the cycle was re‐started by activation of a new action potential in the apex (Fig. 5). As shown in Fig. 5 *A*, we were able to obtain a stable re‐entrant circuit when an activity detected at the base was reinjected at the apex with an imposed time delay of 200 ms. However, when reducing this delay to 150 ms (Fig. 5 *B*), the re‐entrant circuit became unstable, probably due to a subsequent optical stimulus occasionally falling within the refractory phase of the apex. Since in such a case the action potential in the apex failed to propagate to the base, no variation in fluorescence intensity was detected by the system and, consequently, no new optical stimulus was delivered to the apex, effectively interrupting the re‐entrant circuit. In such cases, a spontaneous action potential was required to re‐start the close‐loop manipulation. On average, experiments performed in three different hearts (total number of trials = 30) displayed stable re‐entrant circuits (within a recording time of 16 s) with delays of 200–250 ms while delays of 150–200 ms produced shorter re‐entrant circuits with a lifetime of 6.2 ± 1.9 s. This example demonstrates the capability of the all‐optical platform to simulate re‐entrant circuits in mouse hearts with ease and, more generally, to precisely control its cycle length on demand.

## Discussion

In this work, advances in optogenetics and optics were exploited to implement a wide‐field mesoscope that could map and control the electrical activity of intact mouse hearts in real‐time. The system was characterized in terms of specificity and spatio‐temporal resolution. Custom control software was developed and its ability to operate in closed‐loop control mode with a sub‐2 ms response time was validated in intact ChR2‐expressing mouse hearts, in both healthy and pathological conditions. In the present work, we firstly provided all necessary technical details to reproduce the platform and, secondly, we demonstrated all‐optical closed‐loop control in three different proofs of principle: (i) manipulation of intraventricular electrical activation, (ii) manipulation of AV conduction, and (iii) induction of re‐entrant circuits.

Looking forward, dynamic control of ventricular excitation waves can have a large impact on the study and treatment of arrhythmias. We have previously demonstrated that low‐energy mechanistically based ChR2 stimulation patterns can efficiently terminate cardiac arrhythmias (Crocini *et al*. 2016a), but since that system was not capable of operating in real‐time, cardioversion depended on the inter‐experiment regularity of arrhythmias in our mouse model. In contrast, the closed‐loop control platform presented here could differentially stimulate the tissue based on the timing and route taken by each re‐entrant circuit. Such a capability is essential for the study of the minimum requirements for successful defibrillation in larger animals. Compared to the mouse model system used here, larger animals may tend to display more complex arrhythmias due to their increased ventricular surface area (Garrey, 1914; Winfree, 1994; Vaidya *et al*. 1999), and these may best be cardioverted using simultaneous multi‐site stimulation. Multi‐site optogenetic stimulation is also essential to explore strategies for real‐time resynchronization therapy (Nussinovitch & Gepstein, 2015). In these cases, the real‐time algorithms must be capable of detecting the wave‐front shape and propagation direction and, based on that, design a tailored stimulation pattern. Furthermore, especially for real‐time resynchronization, a closed‐loop frequency of 500 Hz is probably not sufficient. Currently, the maximal closed‐loop frequency was imposed by the frame rate achievable with our camera. Future developments in sCMOS technology, data transfer and storage will likely improve the achievable frame rate. Furthermore, the system could be implemented with a field‐programmable gate array (FPGA) video acquisition card capable of significantly improving reaction time by performing image processing on board.

Methods for real‐time manipulation of excitation waves could be leveraged to develop alternative approaches to study action potential alternans (Merchant & Armoundas, 2012). Action potential alternans are defined as beat‐to‐beat alterations of action potential duration and can occur in either healthy or diseased hearts. Action potential alternans are classified as concordant, when action potential duration shortens or prolongs simultaneously in all cells, or discordant, when neighbouring cells display alternans in opposite phases. Although the cellular mechanisms are not fully elucidated, discordant alternans are known to increase predisposition to arrhythmogenesis in diseased hearts (Ng *et al*. 2007; Narayan *et al*. 2008). The possibility to manipulate the action potential duration optically (Park *et al*. 2014) combined with our optical system could allow a detailed investigation of the level of spatial and temporal discordance required for the onset of an arrhythmic event, as well as assess strategies to control alternans that go beyond current methods, which rely on a single stimulating electrode.

In the present work, we demonstrated the capability of the system in modulating AV delay and treating AV block by monitoring atrial electrical activity. Our system could be employed to investigate rate‐adaptive AV delay modulation. For instance, administration of a β‐adrenergic agonist would increase the heart rate and thus change the optimal AV delay. By using heart rate modulators, our optical platform could be used to optimize AV delay in a variety of conduction disorders. ChR2 expression could be achieved in transgenic models of heart block, brady‐cardiomyopathy or arrhythmia (Fabritz *et al*. 2004; Milan & MacRae, 2005; Baruscotti *et al*. 2011) by means of viral transduction (Vogt *et al*. 2015). The closed‐loop control capability of our platform enabled dynamic responses to changing activity, allowing assessment of real‐time intervention strategies.

While in the first two examples the platform essentially represented a work bench on which various intervention protocols were tested to correct electrical abnormalities, in the third example, the real‐time approach was used to impose a customizable electrical dysfunction. In this case, the possibility to create and control the properties of re‐entrant circuits represents an important achievement that will supply not only basic mechanical insights of this electrical dysfunction but also a reliable model for pharmacological screening.

Functional light‐based technologies for actuation and detection (Crocini *et al*. 2014a, 2017) that provide fundamental insights in cardiac pathophysiology are now proven. The development of an all‐optical control platform provided the first proof‐of‐concept demonstration that optogenetics can be used to dynamically control complex cardiac rhythms based on real‐time detection of excitation waves, opening new research directions for studying cardiac pathophysiology.

## Appendix

All optical, mechanical and electronic details necessary to reproduce the methodology are listed here.

### Optical set‐up

**Figure A1:**
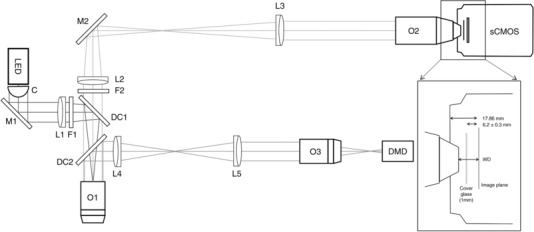
Custom mesoscope C, collimation lens; DC, dichroic mirror; DMD, digital micro‐mirror device; F, filter; L, lens; LED, light emitting diode; M, mirror; O, objective; sCMOS, scientific complementary metal‐oxide semiconductor camera; WD, working distance.

A LED (M625L3, Thorlabs, Newton, NJ, USA; 625 nm centre wavelength, 18 nm full width at half‐maximum (FWHM) bandwidth) is collimated (C, ACL2520U‐A, Thorlabs), bandpass‐filtered (F1, FF01‐640/40‐25 Semrock, Rochester, NY, USA) and focused by a lens (L1, *f* = 100 mm) to generate Köhler illumination at the sample objective (O1, TL2x‐SAP, Thorlabs, ×2, 0.1 NA) (Fig. A1; see Table A1 for a complete list of optical components). A dichroic mirror (DC1, FF685‐Di02‐25x36, Semrock) is used to inject the excitation light (red). The fluorescence (green) emitted by the red‐shifted, electro‐chromic VSD (Di‐4‐ANBDQPQ) is bandpass‐filtered (F2, FF01‐775/140‐25, Semrock) and re‐imaged with two 4f‐lens systems (F2, F3, both *f* = 150 mm) onto the back focal plane of the camera objective (O2, LD Plan‐Neofluar ×20, 0.4 NA, Carl Zeiss Microscopy, Oberkochen, Germany) which creates an image directly onto the chip of a sCMOS camera (OrcaFlash4.0, Hamamatsu, Shizuoka, Japan). The objective has a long working distance (WD) of 7.9 mm (at coverglass of 0.75 mm thickness) which matches the geometrical constraints to reach the chip of the camera embedded in a vacuum protected by a coverglass (see inset Fig. A1). Only a central portion (128 × 128 pixels) of the sensor is used and allows operation at an increased frame rate of 1.6 kHz corresponding to an actual exposure time of 630 μs.

**Figure A2:**
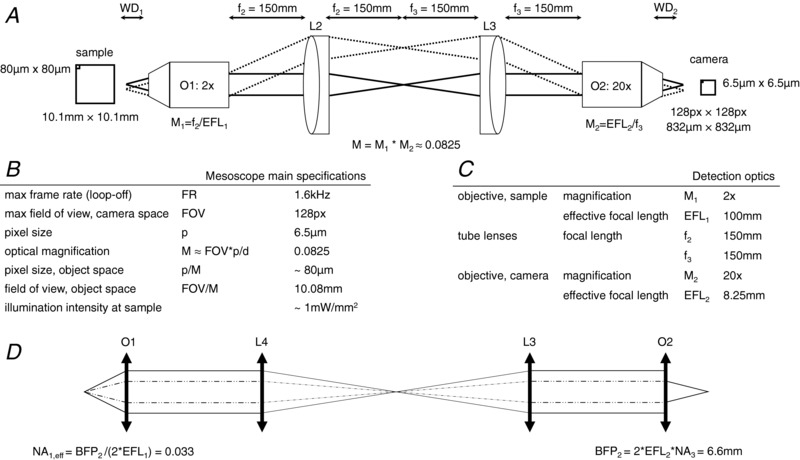
Main considerations for the mesoscope's temporal and spatial resolution BFP, back focal plane diameter; NA, numerical aperture; O, objective; WD, working distance.

**Table A1:** Optical components

Component	Manufacturer	Part no.		Specifications
Collimator	Thorlabs, Newton, NJ, USA	ACL2520U‐A	C	Aspheric condenser lens, ϕ 25 mm, *f* = 20.1 mm, NA = 0.60, ARC: 350–700 nm
Elliptical mirror	Thorlabs	BBE1‐E02	M1	1″ broadband dielectric elliptical mirror, 400–700 nm
	Thorlabs	BBE1‐E03	M2	1″ broadband dielectric elliptical mirror, 750–1100 nm
Lenses	Thorlabs	AC254‐100‐A‐ML	L1	*f* = 100 mm, ϕ 1″ achromatic doublet, ARC: 400–700 nm
	Thorlabs	AC254‐150‐B‐ML	L2	*f* = 150 mm, ϕ 1″ achromatic doublet, ARC: 650–1050 nm
	Thorlabs	AC254‐150‐B‐ML	L3	*f* = 150 mm, ϕ 1″ achromatic doublet, ARC: 650–1050 nm
	Thorlabs	AC254‐100‐A‐ML	L4	*f* = 100 mm, ϕ 1″ achromatic doublet, ARC: 400–700 nm
	Thorlabs	AC254‐100‐A‐ML	L5	*f* = 100 mm, ϕ 1″ achromatic doublet, ARC: 400–700 nm
Dichroic mirrors	Semrock, Rochester, NY, USA	FF685‐Di02‐25x36	DC1	Single‐edge dichroic beamsplitter, edge wavelength: 685 nm long‐pass, 25.2 mm × 35.6 mm × 1.1 mm
	Semrock	FF484‐FDi01‐25x36	DC2	Single‐edge dichroic beamsplitter, edge wavelength: 484 nm long‐pass, 25.2 mm × 35.6 mm × 1.1 mm
Filters	Semrock	FF01‐640/40‐25	F1	Single‐band bandpass filter, centre wavelength: 640 nm, bandwidth (FWHM): 48.9 nm, ϕ 25 mm
	Semrock	FF01‐775/140‐25	F2	Single‐band bandpass filter, center wavelength: 775 nm, bandwidth (FWHM): 146.3 nm, ϕ 25 mm

″, inch; ϕ, diameter; ARC, anti‐reflection coating; *f*, focal length; FWHM: full width at half‐maximum; NA, numerical aperture.

Optogenetic activation (blue) was implemented through a commercially available lightcrafter (DLPLCR4500EVM, Texas Instruments, Dallas, TX, USA) comprising a light engine including RGB LEDs and a 912 × 1140 diamond pixel 0.45 inch WXGA digital micro‐mirror device (DMD). DMD functional principles and specifications are detailed in Fig. 3, while changes made to the lightcrafter to adapt it to its use for optogenetic activation are described in detail in Fig. A4. The primary image projected by the lightcrafter was made to coincide with the detection field of view of a third objective (O3, TL2x‐SAP, Thorlabs, ×2, 0.1 NA) and reimaged onto the sample by means of two 4f‐lens systems (F2, F3, both *f* = 100 mm). The light used for ChR2 activation was injected into the beam path with a second dichroic mirror (DC2, FF484‐FDi0136, Semrock).

### Specifications of the mesoscope

Considering the temporal resolution required to follow action potential propagation (1 ms) and the minimum sampling frequency necessary according to the Nyquist theorem, the mesoscope needs to operate at a frame rate of around 2 kHz corresponding to a frame exposure time of 500 μs. The maximum number of pixel lines the OrcaFlash4.0 sCMOS camera can support in free‐run mode at this approximate frame rate is 128 lines at 1.6 kHz when using camera link. Taking into account a typical mouse heart of approximately 10 mm × 10 mm, the total magnification of the mesoscope has to be 0.0825 where 1 pixel on the camera (6.5 μm) corresponds to approximately 80 μm in the mouse heart and consequently 128 pixels × 128 pixels corresponds to a field of view of approximately 10 mm × 10 mm in the sample plane.

The above considerations on magnification requirements set the focal lengths of both tube lenses used in the two 4f‐systems (Fig. A2) and therefore lead to a mismatch of beam diameters; the clear aperture at the back focal plane of the camera objective (BFP2 = 6.6 mm) imposes a limit to the effective beam diameter at the sample objective and therefore a reduction of its nominal to an effective NA (NA_eff_) of 0.033. Using this effective NA, the optical resolution of the two 4f‐lens systems in terms of the FWHM of its point spread function (PSF) is 0.61 × λ × *M*/NA_eff_ = 0.8 μm when using light of λ = 500 nm and an optical magnification M = 0.0825.

In closed‐loop mode, 8‐bit images are saved to a solid‐state disk (SSD) at 500 Hz where a custom‐made LabVIEW program (National Instruments, Austin, TX, USA) performs a real‐time image analysis. While images could also be buffered and processed in the workstation's random access memory (RAM) to increase the 2 ms temporal resolution, saving to the SSD allows us to use proprietary Hamamatsu software for time lapse acquisition and a simple LabVIEW program for real time analysis.

### Digital micro‐mirror device

**Figure A3:**
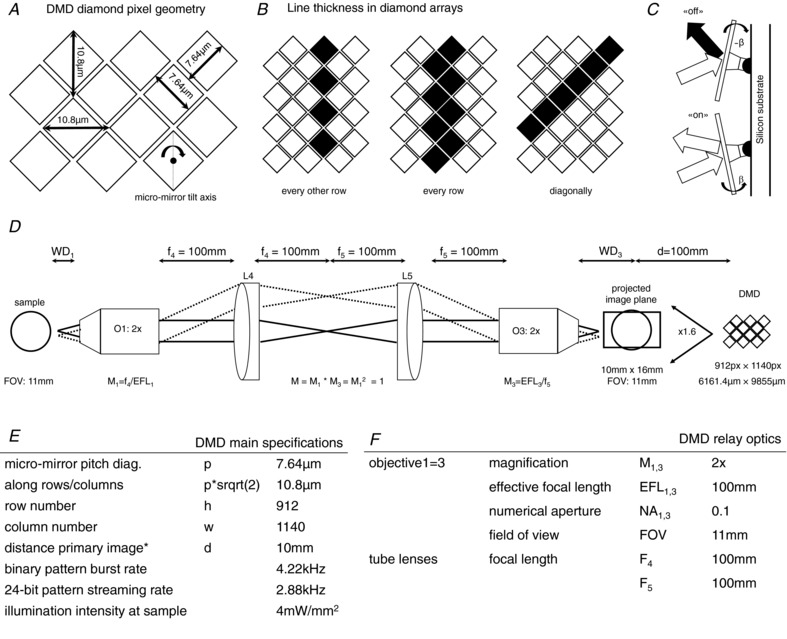
Specifications of the digital micro‐mirror device (DMD) and its relay optics after removing the projection lens

The mirrors on the digital micro‐mirror device (DMD) are arranged on a diamond pixel geometry (Fig. A3) with a mirror pitch of 7.64 μm and 10.8 μm along the diagonal and orthonormal directions, respectively (see also table in Fig. A3 *E*). Lines can be projected in three different geometries ranging from thinnest when using every other row (or column) over pixel‐width when using diagonals to broadest when using each row (or column) (Fig. A3 *B*). By applying a tilt angle of β = 12 degrees, each micro‐mirror can be switched on or off individually (Fig. A3 *C*). The relay optics used to project the DMD pattern onto the sample (Fig. A3 *D*) combine to a 1:1 re‐imaging system by using two identical objectives with two identical respective tube lenses (Fig. A3 *F*). When removing the projection lens from the light crafter as described in Fig. 4, the DMD projects a primary image of approximately 10 mm × 16 mm at a distance *d* of 10 mm from the front housing of the light crafter. Considering the active area of 6161.4 μm × 9855 μm, the projection results in a 1.6 magnification. The personal computer (PC) controlling the lightcrafter is equipped with a graphics card that has two HDMI output ports. One is used for the PC screen, and the other is used to send activation patterns to the lightcrafter. See Table A2 for a full list of hardware components and Fig. A6 for details of electronic connectivity.

**Figure A4:**
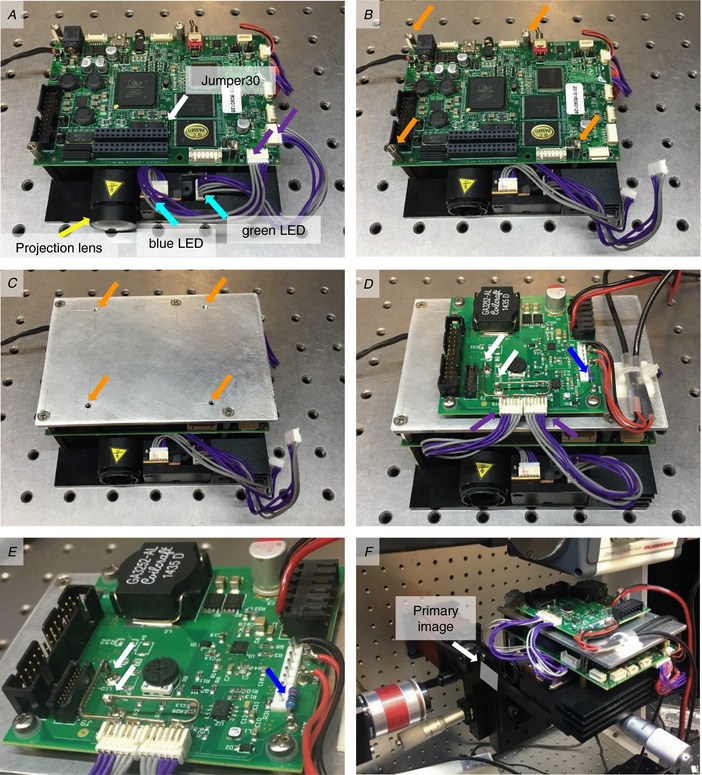
The lightcrafter as optogenetic stimulation light source *A*, remove the projection lens (yellow arrow) by gently wedging it out of its mount with a screwdriver. To bypass the native LED driver, populate Jumper 30 (white arrow). Unplug the connectors (purple arrows) for the blue and green LEDs (cyan arrows). *B*–*D*, using spacers (orange arrows, *B*) mount an aluminium plate with adequately spaced and threaded through holes (*C*) to mount the external LED driver (*D*). *E*, on the LED driver, mount connectors for the blue and green LED and connect them to the LED+ and LED− terminals (white arrows) in a series configuration and solder a pull‐up resistor (blue arrowhead). *F*, the lightcrafter is mounted on a manual *xyz* tip stage which is approximately inclined by 10 degrees. Without the projection lens, the lightcrafter projects a primary image (white arrow) approximately 10 mm from the front housing.

**Table A2:** Hardware components

Component	Manufacturer	Part no.	Specifications
LED	Thorlabs, Newton, NJ, USA	M625L3	625 nm centre wavelength, 18 nm FWHM bandwidth, 700 mW, 1000 mA
Objectives	Thorlabs	TL2x‐SAP	×2, 0.1 NA, 56.3 mm WD, 100 mm EFL (sample and light crafter)
	Zeiss, Oberkochen, Germany	421350‐9970‐000	×20, 0.4 NA, 7.9 mm WD, 8.25 mm EFL, coverslip correction for 0.75 mm (camera)
Camera	Hamamatsu Photonics, Shizuoka, Japan	OrcaFlash4.0 v2.0	sCMOS sensor, 2048 × 2048, pixel size: 6.5 μm, 16‐bit images
Lightcrafter	Texas Instruments, Dallas, TX, USA	DLPLCR4500EVM	912 × 1140 diamond pixel 0.45 inch WXGA DMD, RGB LED light engine, two configurable I/O triggers, video display up to WXGA resolution (1280 × 800)
LED driver board	Texas Instruments	LM3433SQ‐ 4AEV/NOPB	9–14 V, 20 A
DAQ boards	National Instruments	NI USB‐6212	AI: 400 kS s^−1^ multichannel; 16‐bit resolution; AO: 250 kS s^−1^, 16‐bit resolution; 24 digital I/O lines; two counters, 32‐bit; 80 MHz max counter frequency
		NI USB 6002	AI: 50 kS s^−1^ multichannel; 16‐bit resolution; AO: 5 kS s^−1^, 16‐bit resolution; 13 digital I/O lines; one counter, 32‐bit; 5 MHz max counter frequency
Workstation	Dell	Precision Tower 5810	32 GB RAM, Intel Xeon processor E5‐1630 v3 at 3.70 GHz, OS Windows 7 Professional 64‐bit
Graphics card	Nvidia, USA	Quadro K620	2 GB DDR3 GPU memory, 128‐bit memory interface, 29 GB s^−1^ memory bandwidth, PCI Express 2.0 × 16 interface, DVI‐D DL + DP 1.2 display connectors
El. stimulator	Digitimer, UK	DS2A‐Mk II	99 V output, low noise battery power supply, int. (20 μs to 2 s) or ext. TTL control of pulse duration
ECG ampliier	A‐M Systems Inc., USA	3000 AC/DC differential amplifier	Noise: 1.8 μV, p‐p (10 Hz to 10 kHz); 0.1 fA Hz^−1^ at 1 kHz; input impedance: 10^15^ Ohms/1 pF; initial bias current: ±1.0 fA
Power supplies	MeanWell, Taiwan	T‐60B	For lightcrafter, 12 V output voltage, 5 A output current, 60 W output power
	RS components, UK	RS‐150‐12	For external LED driver board; 12 V output voltage, 12.5 A output current, 150 W output power
Mechanical stages	Thorlabs	MLJ150‐M	motorized *z*‐stage
		MT‐FN1	manual *xy*‐stage

DMD, digital micro‐mirror device; DVI, digital visual interface; EFL, effective focal length; FWHM, full width at half‐maximum; GPU, graphics processing unit; I/O, input/output; LED, light emitting diode; NA, numerical aperture; PCI, peripheral component interconnect; RAM, random access memory; RGB, red green blue; SCMOS, scientific complementary metal‐oxide semiconductor; TTL, transistor transistor logic; WD, working distance.

#### Spatial resolution

Assuming a diagonal pixel line of 7.64 μm width on the DMD, the resulting projected line in the primary imaging plane has a width of 12.6 μm. Using a wavelength of λ = 455 nm, unit magnification and an NA of 0.1, the FWHM of the PSF of the re‐imaging system is PSF (FWHM) = 0.61 × λ × *M*/NA = 2.78 μm. This means that the spatial resolution of the system is currently limited by the micro‐mirror size of the DMD.

#### Temporal resolution

The DMD can be operated in two different modes, streaming and burst mode. In streaming mode, the DMD receives continuous 24‐bit patterns from the parallel RGB interface and streams them at 2.88 kHz (data rate: 2.99 Gb s^−1^). In burst mode, 48 binary patterns are loaded from flash storage into internal memory and can be displayed at 4.22 kHz (data rate: 4.39 Gb s^−1^).

#### Optical efficiency

Averaging over different activation patterns, the power coupling efficiency of the relay optics was measured as 32.7 ± 0.8%. Using the LED driver at the maximum permissible current (sum of all three LED currents must be less than 4.3 A at all times), in the focal plane of objective1 (sample plane) we obtain a maximum intensity of 4 mW mm^−2^.

### Modifications to the lightcrafter

In its native state, the RGB LEDs in the lightengine of the lightcrafter (DLPLCR4500EVM, Texas Instruments) are commanded by an internal LED driver that uses a reduction of the duty cycle to dim light intensity. To circumvent dimming unsuitable for ChR2 excitation, an external LED driver board (LM3433SQ‐14AEV/NOPB) was implemented into the lightcrafter. A complete list of hardware components can be found in Table A2.

**Figure A5:**
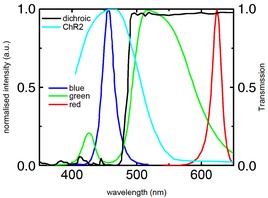
Emission spectra of the red green and blue LEDs in the light engine Spectrum is indicative only. Also shown is the transmission curve of the dichroic filter (black) used for injecting the optogenetic excitation light into the mesoscope beam path and the excitation spectrum of ChR2 (cyan).

In the following, the procedure to adapt the lightcrafter for use as an optogenetic stimulation source and to change the LED driver board is described (see Fig. 4). Firstly, remove the projection lens (yellow arrow, Fig. 4 *A*) by gently wedging it out of its mount with a screwdriver. Secondly, to bypass the native LED driver, populate jumper J30 (white arrow, Fig. 4 *A*), then proceed to disconnect the supply connectors (purple arrows, Fig. 4 *A*) for the blue and green LEDs (cyan arrows, Fig. 4 *A*). Due to the wide emission spectra of the green LED, light intensity within the ChR2 excitation spectrum was increased by taking advantage of the green tail inside the ChR2 excitation spectrum (see also Fig. A5, this document). By using appropriate spacers (orange arrows, Fig. 4 *B* and *C*) and an aluminium plate, thirdly, mount the external LED driver board on top of the lightcrafter.

The following modifications need to be made to the LED driver board. First, solder new supply connectors for the blue and green LEDs by connecting the cathode of LED1 to the LED− pin, the anode of LED1 to the cathode of LED2 and the anode of LED2 to the LED+ pin on the external LED driver board. Second, solder the cables to connect the LEDs and the driver board to external power supplies. A pull‐up resistor (blue arrows, Fig. 4 *D* and *E*) was used to ensure a well‐defined off‐state when the driver was uncoupled. Mount the lightcrafter on a manual *xyz* stage with additional tip movement since an inclination of 10 degrees away from the optical axis is needed to compensate for the intrinsic upwards projection of the lightcrafter. Without the projection lens, the lightcrafter projects an image at a distance of 15 mm. This image (approx. 10 mm × 16 mm) was aligned to coincide with the detection field of view of the objective (Fig. 4F).

### Spectra of the light engine

Figure 5 shows the emission spectra of the three LEDs in the light engine of the lightcrafter. Luminosity within the excitation spectrum of ChR2 (cyan) was increased by using not only the blue LED but also taking advantage of the green tail peaking at approximately 425 nm with a FWHM of approximately 25 nm. All spectra are indicative only. Also shown is the relative transmission of the dichroic long‐pass filter (black, FF484‐FDi01, Semrock) used to inject the optogenetic excitation light into the mesoscope beam path. Table A3 lists the LED emission peak wavelength and the spectrum bandwidth (FWHM).

**Table A3:** LED parameters

LED	Centre (nm)	Bandwidth (nm)
Blue	455	27
Green	520	100
Red	624	18

### Electronic connectivity scheme

**Figure A6:**
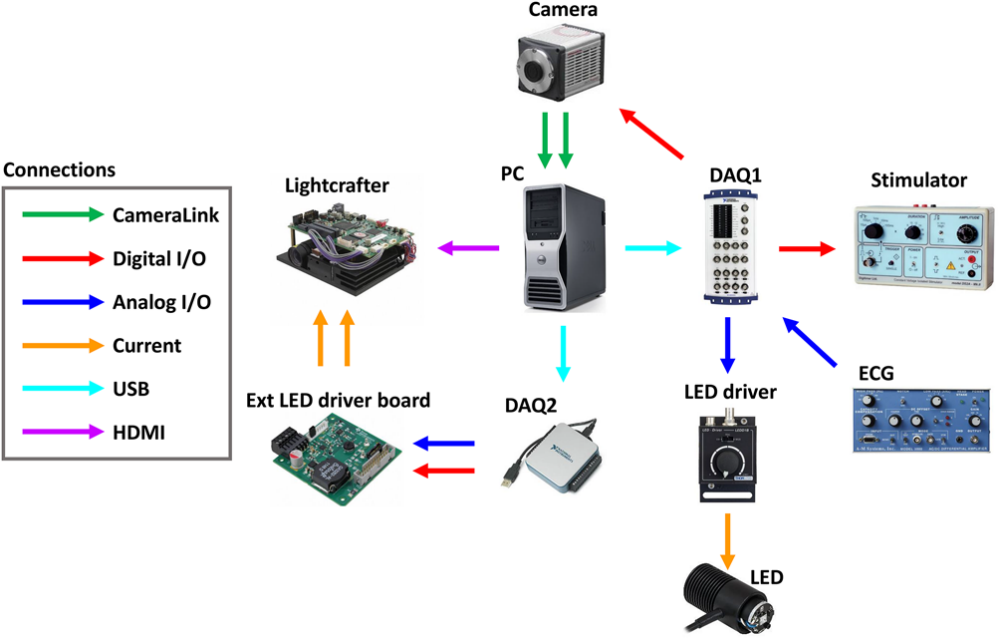
Connectivity scheme of the mesoscope DAQ, data acquisition device; ECG, electrocardiogram; ext, external; HDMI, high‐definition multimedia interface; I/O, input/output; LED, light emitting diode; USB, universial serial bus; PC, personal computer.

As shown in Fig. A6, the mesoscope is operated via two data acquisition devices (DAQ1: NI USB‐6212 and DAQ2: NI USB 6002, National Instruments), which are both connected to a PC via universal serial bus (USB). DAQ1 provides an analog output (AO), which regulates the current in the light emitting diode (LED) driver and therefore LED emission power. Further, DAQ1 receives an analog input (AI) from the ECG. A digital output (DO) from DAQ1 provides an external trigger to the camera and a programmable free interface (PFI) is used to trigger the stimulator. DAQ2 supplies two outputs to the external LED driver board mounted on the lightcrafter: an analog channel supplies the current and therefore modulates LED emission power in the light engine while a digital channel acts as a shutter. The camera is connected via a camera link to an internal card inside the PC. The lightcrafter is commanded via HDMI from the graphics card inside the PC. See Table A2 for a complete list of hardware components and their technical specifications.

## Additional information

### Author's present address

C. Crocini: Department of Molecular, Cellular, and Developmental Biology and BioFrontiers Institute, University of Colorado, Boulder, CO, USA.

### Competing interests

The authors declare no conflict of interest.

### Author contributions

M.S., C.M.: acquisition, analysis and interpretation of data, drafting the manuscript. E.M., S.C., R.C., P.Y. and M.C.: acquisition, analysis and interpretation of data. C.C.: drafting the manuscript. C.F., L.M.L., L.B., D.G., E.C., C.P. and F.S.P.: revising the work critically for important intellectual content. G.B.: design of the work, revising it critically for important intellectual content. L.S.: design of the work, drafting the manuscript. All authors have read and approved the final version of this manuscript and agree to be accountable for all aspects of the work in ensuring that questions related to the accuracy or integrity of any part of the work are appropriately investigated and resolved. All persons designated as authors qualify for authorship, and all those who qualify for authorship are listed.

### Funding

This work was supported by the European Union Horizon 2020 research and innovation program under grant agreement no. 654148 Laserlab‐Europe, by National Institutes of Health (NIH Grant: R01 EB001963), by the Italian Ministry for Education, University and Research in the framework of the Flagship Project NANOMAX, by the Italian Ministry of Health (WFR GR‐2011‐02350583), by Telethon–Italy (GGP13162), by Ente Cassa di Risparmio di Firenze (private foundation), and by FAS‐Salute ToRSADE project. C.C. holds a long‐term fellowship from the Human Frontiers Science Program Organization.

## Supporting information

Figure S1. Cross‐talk free optogenetic stimulation during VSD imaging.
Figure S2. Dose‐response curves to different ChR2 stimulation patterns designed as reported by the blue ROIs on the fluorescence image of the heart.
Figure S3. VSD signal‐to‐noise ratio and threshold selection.
Figure S4. Optical manipulation of atrioventricular delay.
