# Supplementary material for: Real‐time optical manipulation of cardiac conduction in intact hearts
Source: J Physiol. 2018 Aug 7;596(17):3841–58. doi: 10.1113/JP276283 (PMC6117584; doi:10.1113/JP276283)
Supplement: Supplementary file 1 — Figure S1. Cross‐talk free optogenetic stimulation during VSD imaging. Figure S2. Dose‐response curves to different ChR2 stimulation patterns designed as reported by the blue ROIs on the fluorescence image of the heart. Figure S3. VSD signal‐to‐noise ratio and threshold selection. Figure S4. Optical manipulation of atrioventricular delay. [file TJP-596-3841-s001.pdf]

SUPPLEMENTARY FIGURES

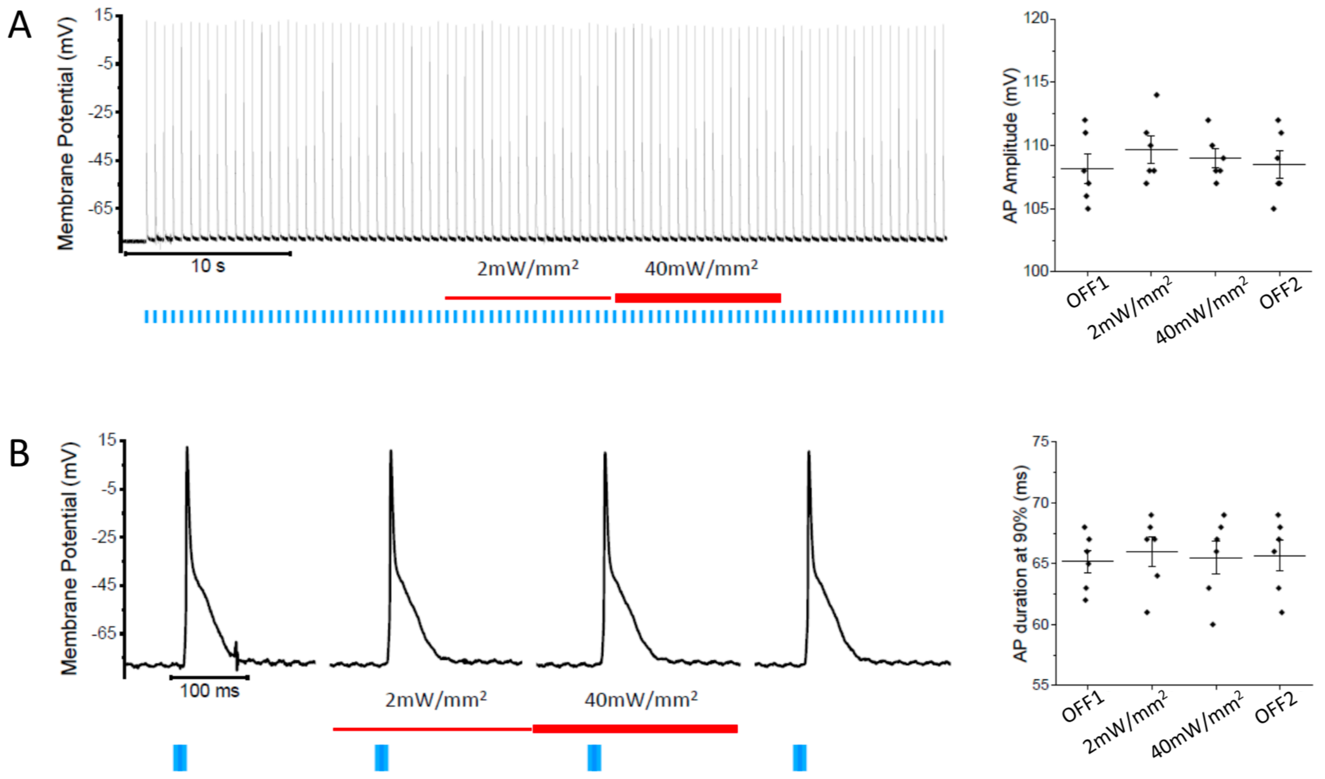

Figure S1: Cross-talk free optogenetic stimulation during VSD imaging. On the left, a representative trace of membrane potential recorded in cardiomyocytes isolated from ChR2 heart using patch clamp. Action potentials were induced using blue light pulses (blue ticks). During stimulation, the red LED used for VDS imaging was turned on with two different power intensities (2 mW/mm^2, thin red line and 40 mW/mm^2, thick red line) showing no variation in action potential amplitude (A) or duration (B). OFF1 and OFF2 represent phases before and after red LED illumination respectively. On the right, action potential amplitude (A) and duration (B) during different states of red LED illumination. Each data point (black dot) represents the average measured in an individual cell. Also indicated is the mean and the standard error of the mean of each illumination state. No statistically significant differences (Student's t-test, number of cells = six) were found between categories.

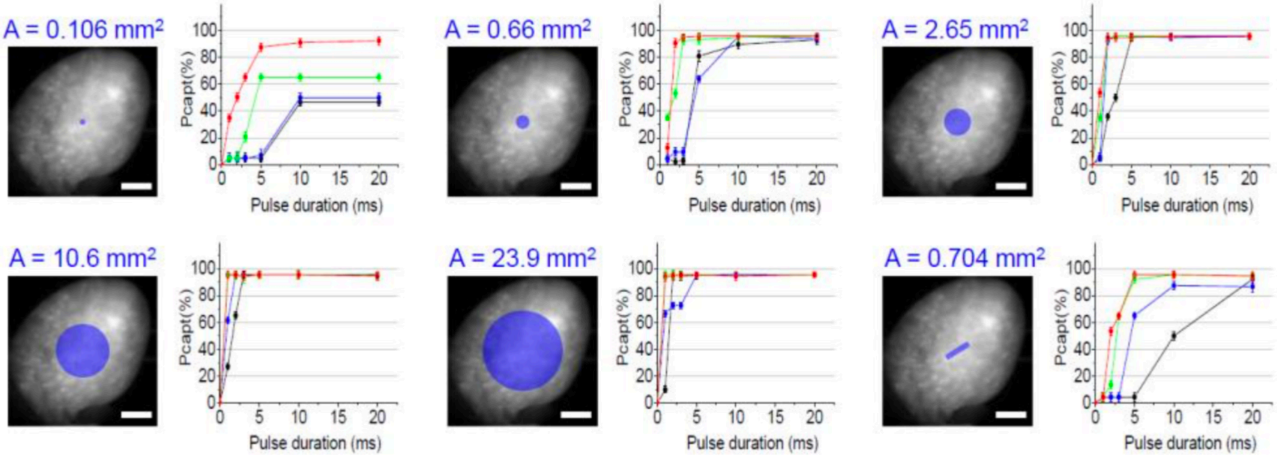

Figure S2: Dose-response curves to different ChR2 stimulation patterns designed as reported by the blue ROIs on the fluorescence image of the heart. The area associated to each pattern is reported above the images. Capture probability (Pcapt) for each ChR2 stimulation pattern in function of pulse duration and LED intensity while keeping a constant number of 10 pulses at a frequency of 5 Hz. Pcapt is defined as the percentage of all flashes given that successfully captured the ventricle. Different colours depict different LED intensities: black = 1 mW/mm^2, blue = 1.7 mW/mm^2, green = 3 mW/mm^2 and red = 4.3 mW/mm^2. Data reported as mean ± SEM (standard error of the mean) from N = 6. Scale bar of 2 mm in white.

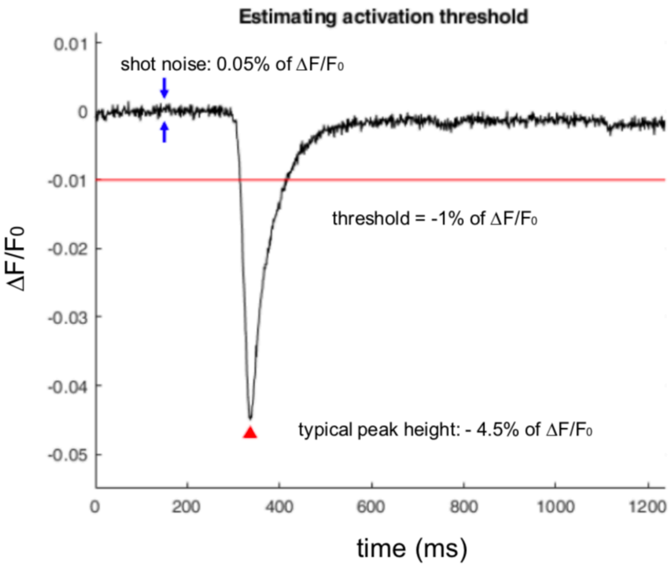

Figure S3: VSD signal-to-noise ratio and threshold selection. Typical $\Delta F/F_0$ trace extracted during real-time operation from a ROI containing approximately 400 pixel^2 showing shot noise of 0.05% of $\Delta F/F_0$ (standard deviation of trace at rest) and a peak height of -4.5% of $\Delta F/F_0$ corresponding to the expected sensitivity of the VSD of the order of 4%. Considering the ratio of shot noise to peak-height, a threshold of -1% of $\Delta F/F_0$ was selected to trigger action potential detection.

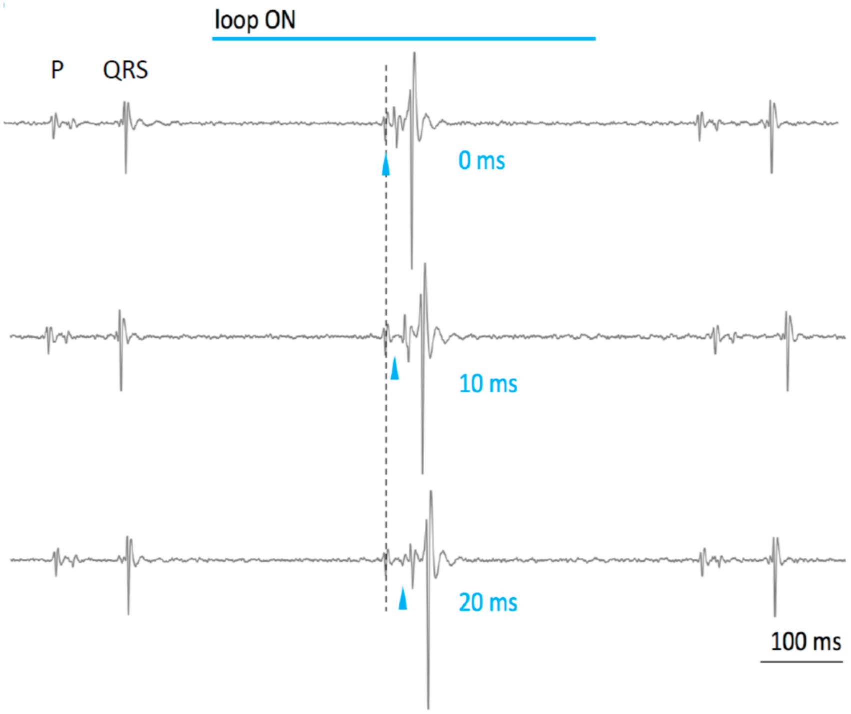

Figure S4: Optical manipulation of atrioventricular delay. ECG for the three applied delays shown in Fig 4. Blue arrowheads highlight the ChR2 stimulation performed using the single-point activation pattern.
